# Balancing Thymocyte Adhesion and Motility: A Functional Linkage Between β1 Lntegrins and The Motility Receptor RHAMM

**DOI:** 10.1155/2000/94616

**Published:** 2000

**Authors:** Sheryl L. Gares, Linda M. Pilarski

**Affiliations:** 1 Department of Oncology University of Alberta Cross Cancer Institute Edmonton AB Canada; 2 Department of Oncology Cross Cancer Institute 11560 University Ave. Edmonton, AB T6G 1Z2 Canada

**Keywords:** adhesion, β1 integrins, hyaluronan, motility, RHAMM, thymocytes

## Abstract

Thymocyte differentiation involves several processes that occur in different anatomic sites within the thymus. Therefore, thymocytes must have the ability to respond to signals received from stromal cells and adopt either adhesive or motile behavior. We will discuss our data indicating human thymocytes use α4β1 integrin, α5β1 integrin and RHAMM to mediate these activities. Immature multinegative (MN; CD3–4–8–19-) thymocytes use α4β1 and α5β1 integrins to mediate weak and strong adhesion. This subset also uses α4β1 integrin to mediate motility. As thymocytes differentiate, they begin to express and use RHAMM to mediate motility in conjunction with α4β1 and α5β1 integrins. Motile thymocytes use β1 integrins to maintain weakly adhesive contacts with substrate to provide traction for locomoting cells, thus weak adhesion is a requirement of motile behavior. Hyaluronan (HA) is also required by thymocytes to mediate motility. HA binding to cell surface RHAMM redistributes intracellular RHAMM to the cell surface where it functions to mediate motility. We propose that the decision to maintain adhesive or motile behavior is based on the balance between low and high avidity binding conformations of β1 integrins on thymocytes and that RHAMM:HA interactions decrease high avidity binding conformations of integrins pushing the balance toward motile behavior.


*Developmental Immunology*, 2000, Vol. 7(2-4), pp. 209-225
Reprints available directly from the publisher
Photocopying permitted by license only

© 2000 OPA (Overseas Publishers Association)
N.V. Published by license under the
Harwood Academic Publishers imprint,
part of the Gordon and Breach Publishing Group.
Printed in Malaysia

### Balancing Thymocyte Adhesion And Motility: A Functional Linkage Between β1 Integrins and the Motility Receptor RHAMM

SHERYL L. GARES and LINDA M. PILARSKI*

*Department of Oncology, University of Alberta and Cross Cancer Institute, Edmonton, AB, Canada*

Thymocyte differentiation involves several processes that occur in different anatomic sites within the thymus. Therefore, thymocytes must have the ability to respond to signals received from stromal cells and adopt either adhesive or motile behavior. We will discuss our data indicating human thymocytes use α4β1 integrin, α5β1 integrin and RHAMM to mediate these activities. Immature multinegative (MN; CD3–4–8–19-) thymocytes use α4β1 and α5β1 integrins to mediate weak and strong adhesion. This subset also uses α4β1 integrin to mediate motility. As thymocytes differentiate, they begin to express and use RHAMM to mediate motility in conjunction with α4β1 and α5β1 integrins. Motile thymocytes use β1 integrins to maintain weakly adhesive contacts with substrate to provide traction for locomoting cells, thus weak adhesion is a requirement of motile behavior. Hyaluronan (HA) is also required by thymocytes to mediate motility. HA binding to cell surface RHAMM redistributes intracellular RHAMM to the cell surface where it functions to mediate motility. We propose that the decision to maintain adhesive or motile behavior is based on the balance between low and high avidity binding conformations of β1 integrins on thymocytes and that RHAMM:HA interactions decrease high avidity binding conformations of integrins pushing the balance toward motile behavior.

*Keywords:* adhesion, β1 integrins, hyaluronan, motility, RHAMM, thymocytes

*Abbreviations:*DN , double negative, DP, double positive, ECM, extracellular matrix, FAK, focal adhesion kinase, GAG, glycosaminoglycan, Fn, fibronectin, HA, hyaluronan, HAS, hyaluronan synthase, MN, multinegative, RHAMM, receptor for hyaluronan-mediated motility, SP, single positive, TCR, T cell receptor

### INTRODUCTION

Development of a diverse peripheral T cell repertoire able to respond to a broad array of potential pathogens, yet tolerant to self depends upon a complex, orchestrated series of events that occur in the thymus (for recent reviews see Sebzda et al., 1999; Rodewald & Fehling, 1998; Fehling & von Boehmer, 1997; Killeen at al., 1998; von Boehmer & Fehling, 1997). Generation and modeling of the T cell repertoire begins as thymocyte precursors arrive from the bone marrow, begin to rearrange T cell receptor (TCR)

* Corresponding Author: Dr Linda M. Pilarski, Department. of Oncology, Cross Cancer Institute, 11560 University Ave. Edmonton, AB, Canada T6G 1Z2. Telephone: (780) 432 8925, Fax: (780) 432 8928, email: lpilarsk@gpu.srv.ualberta.ca genes and proliferate. Successful TCR gene rearrangement is the first critical step in the development of a functional T cell. Once TCRαβ genes are rearranged and expressed along with CD3, CD4 and CD8, thymocytes undergo positive and negative selection before emigrating the thymus to populate the peripheral immune system. The outcome of positive and negative selection (survival or death) is dependent on signals generated when TCR heterodimers interact with MHC molecules on thymic stromal cells (Amsen & Kruisbeek, 1998; Anderson et al., 1996). It is well recognized that the TCR, MHC class I and II, CD4 and CD8 molecules play a critical role in these interactions (Ellmeier et al., 1999; Sebzda at al., 1999; Chan et al., 1998; Hedrick & Sharp, 1998; Marrack & Kappler, 1997). However, interactions of developing thymocytes with thymic stromal elements; epithelial cells, dendritic cells, fibroblasts and thymic macrophages, also rely upon contacts being made between additional receptors expressed on thymocytes and stromal cells during sessile phases of differentiation (Amsen & Kruisbeek, 1998; Lagrota-Cândido et al., 1996; Dardenne & Savino, 1994; Savino et al., 1993; Ritter & Boyd, 1993; Boyd et al., 1993; Watt et al., 1992; Haynes, 1986). Although the precise path taken by thymocytes as they undergo selection is still unclear, migration must occur within the thymic lobule as thymocytes differentiate; therefore, motility receptors are also required during migratory phases of differentiation. β1 integrins mediate adhesion and motility for human thymocytes (Gares et al, 1998; Salomon et al., 1997; Crisa et al., 1996; Salomon et al., 1994). The receptor for hyaluronan (HA)-mediated motility (RHAMM) also mediates thymocyte motility and may function in conjunction with β1 integrins (Gares et al., 1998; Pilarski et al., 1993). Recently another layer of complexity has been added, since chemokines and chemokine receptors have been found to be expressed in the thymus and to mediate thymocyte chemotaxis. This indicates the mechanisms used by thymocytes to migrate through the thymus are likely to be as complex as those required for the process of selection. The emphasis of this article is to present our data and propose a model that addresses how thymocytes may achieve this task. Our data indicates β1 integrins and RHAMM have a functional relationship within which their expression and function is balanced between adhesion and de-adhesion to ultimately promote either adhesive or motile behavior.

### TRAFFICKING IN THE THYMUS

### Thymic Structure

The thymus is composed of a number of encapsulated lobules which consist of a subcapsular region, a cortex heavily populated with immature, double positive, (DP; $CD4^{+}8^{+}$) thymocytes, nurse cells, macrophages and epithelial cells and a medulla containing macrophages, dendritic cells, medullary epithelial cells and more mature, single positive, (SP; $CD4^{+}8^{-}$ and $CD4^{-}8^{+}$) thymocytes (Boyd et al., 1993; Haynes, 1986; 1984). The medulla also contains thymocytes with the phenotype expected of a generative thymocyte lineage (Pilarski, 1993). Phenotypic analysis of thymocytes and thymic epithelial cells has been used to determine where selective events are likely to occur in order to track the path of differentiating thymocytes (van Ewijk et al., 1994; Boyd et al., 1993; Pilarski, 1993; Boyd & Hugo, 1991; Nikolic-Zugic, 1991; Haynes, 1986). In both mouse and human tissue, the site of entry of pro-thymocytes has not been definitively determined and may be the cortico-medullary junction or the subcapsular region. In addition to an analysis of surface phenotype of thymocytes in the cortex, *in situ* assessment of apoptosing cells in MHC-deficient mice indicates this region is likely to be the anatomic site of positive selection (Surh & Sprent, 1994). Thymocytes that are presumably receiving negative signals can be identified in the medulla, but these results do not exclude a model in which negative selection occurs in the cortex and cortico-medullary junction, with the consequences of negative selection (apoptosis) occurring in the medulla. It is likely positive and negative selection are ongoing processes in which each differentiating thymocyte makes a series of contacts with a variety of

MHC-peptide complexes on numerous stromal cells before a thymocyte is able to fully mature. This model of differentiation emphasizes the requirement for thymocytes to rapidly respond to microenvironmental cues by regulating the function of adhesion and motility receptors while they are within one compartment of the thymus lobe as well as when they migrate to an anatomically distinct site within the thymus.

Extracellular matrix (ECM) components within the human thymus include fibronectin (Fn), laminin, collagens, glycosaminoglycans (GAGs) (Savino et al., 1993; Berrih et al., 1985) and merosin (Chang et al., 1993). In addition to the contacts made between thymocytes and stromal cells, these constituents likely play a role during thymocyte differentiation by maintaining adhesive contacts during sessile phases of development and promoting motility during migratory phases of development. Fn mediates proliferation and adhesion for murine thymocytes (Halvorson et al., 1998; Sawada et al., 1992; Utsumi et al., 1991) and both adhesion and motility for human thymocytes via $\alpha4\beta1$ and $\alpha5\beta1$ integrins (Gares et al, 1998; Salomon et al., 1997; Crisa et al., 1996; Salomon et al., 1994), whereas merosin mediates only adhesion of mouse and human thymocytes via $\alpha6\beta1$ integrin (Chang et al., 1993). Neither we, nor others have found thymocytes adhere to laminin-, vitronectin-, or collagen-coated surfaces. HA is a ubiquitously expressed GAG of the ECM and is a large polysaccharide consisting of repeating dimers of glucuronate and N-acetylglucosamine. HA plays a structural role in ECM providing turgor pressure in tissue spaces and acts as a cushion in joints, but smaller fragments of HA have been shown to mediate a number of cellular effects upon interaction with HA binding proteins or hyaladherins (for reviews see Borland et al., 1998; Fraser et al., 1997; Toole, 1997; Entwistle et al., 1996; Sherman et al., 1994; Knudson & Knudson, 1993). HA mediates motility, endocytosis, signal transduction and cytokine secretion upon interaction with the hyaladherins RHAMM and CD44. Human thymocytes express both RHAMM and CD44, but RHAMM:HA interactions appear to be predominant in the motile process.

### $\beta1$ Integrin-mediated Adherence and Motility

The expression of $\beta1$ integrins in other developing tissues suggested there might be a correlation of integrin expression and function with stages of thymocyte differentiation. Immunohistochemical staining of human thymus as well as staining of thymocyte suspensions and thymic epithelial cultures indicates a number of adhesion receptors are expressed on both cell types with especially strong representation of $\beta1$ and $\beta2$ heterodimers (Watt et al., 1992). Immunohistochemical analysis of human thymus also indicates collagens, Fn and laminin are distributed within the thymus (Berrih et al., 1985; Savino et al., 1993). In vitro experiments indicate ECM constituents influence the growth of thymic epithelial cells, thus are "active" components of the thymic microenvironment. Analysis of the adhesion of murine double negatives (DN; $CD4^-8^-$) indicates these thymocytes spontaneously adhere to Fn-coated plates and to Fn-treated stromal cell monolayers utilizing $\alpha4$ predominantly and $\alpha5$ integrin to a lesser extent (Sawada et al., 1992; Utsumi et al., 1991). Antibody or peptide blocking of these interactions inhibits *in vitro* differentiation of DN thymocytes to the DP stage. Further subset analysis indicates $\alpha4$ expression decreases as thymocytes mature to the DP and SP stages. This work indicates differentiating thymocytes express constitutively active conformations of $\beta1$ integrins and suggests integrins play a role in differentiation. Multinegative (MN; $CD3^-4^-8^-19^-$) human thymocytes purified by negative selection methods contain a similar proportion of Fn-adherent cells and Mabs to $\alpha4$, $\alpha5$ and $\beta1$ block adherence, again indicating $\alpha4\beta1$ and $\alpha5\beta1$ integrins mediate interaction of immature thymocytes with thymic stroma or ECM components (Gares et al., 1998). Although murine DP and SP thymocytes downregulate $\alpha4$ integrin expression, human thymocytes continue to express and utilize $\alpha4\beta1$ as well as $\alpha5\beta1$ as they undergo differentiation. Fn-adherent thymocytes utilizing $\alpha4\beta1$ have a phenotype consistent with DP cells that have not yet received a positive selection signal ($CD4^{hi}8^{hi}3^{med}69^{lo}$) (Salomon et al., 1994). Non-adherent thymocytes consistently contain DP

thymocytes with a "post-selection" phenotype ($CD4^{med}8^{med}3^{hi}69^{hi}$) indicating the non-adherent fraction contains more mature cells. However, α4 expression of these thymocytes is equivalent to that of less mature DP thymocytes, suggesting lack of adhesive behavior is a result of downregulation of α4 function. These experiments indicate a correlation of integrin function with thymocyte differentiation and led to a model proposing that α4β1 facilitates interaction of thymocytes with cortical epithelial cells during positive selection. One of the consequences of positive selection appears to be an alteration of α4β1 to an inactive conformation causing de-adherence enabling thymocyte migration to the medulla.

The α4β1 and α5β1 integrins also function as mediators of motility for human thymocytes (Gares et al., 1998; Crisa et al., 1996). MN thymocytes utilize α4β1 to mediate motility and more mature DP and SP thymocytes use a combination of α4β1, α5β1 and RHAMM to mediate motility. This interplay of integrins and RHAMM will be more fully discussed in subsequent sections.

### Chemokines and Chemokine Receptors

In the last two years a number of chemokines and chemokine receptors have been detected in both murine and human thymus tissue. To date, thymocytes express the chemokine receptors CCR3, CCR5, CCR7, CXCR4 and GPR-9–6 (proposed name CCR9) and expression and function of these receptors correlates with developmental subsets of thymocytes. This suggests chemokines and chemokine receptors play a role in trafficking through the thymus that is likely related to selection signals received during differentiation.

The orphan chemokine receptor GPR-9–6 has been characterized as the receptor for the thymus expressed chemokine TECK (Zaballos et al., 1999). GPR-9–6 mRNA is expressed in immature and mature human and murine thymocytes and cell lines transfected with GPR-9–6 are chemotactic in response to TECK. Thymic dendritic cells produce TECK, so it is speculated that GPR-9–6 may be utilized by thymocytes through all stages of differentiation to mediate chemotaxis. CXCR4 is also expressed on immature $CD34^+TCR^-$ human thymocytes (Zaitseva et al., 1998) and mRNA transcripts encoding murine CXCR4 are detected among the immature $CD44^+25^+4^-8^-$ subset (Suzuki et al., 1999). CXCR4 expression is downregulated on more mature SP subsets. The ligand of CXCR4, stromal cell-derived factor-1 (SDF-1) mediates $Ca^{2+}$ flux and chemotaxis of immature DN and DP thymocytes and is expressed by cortical stromal cells suggesting a role for this chemokine in early thymocyte differentiation

CCR5 is weakly expressed by human thymocytes with the exception of the $CD34^+TCR^-$ subset, which shows high expression of both CXCR4 and CCR5 (Zaitseva et al., 1998). However, this group did not find CCR5 ligands (RANTES, MIP-1α, MIP-1β) stimulated chemotaxis or $Ca^{2+}$ flux of human thymocytes. Another group reported MIP-1β supported chemotaxis of all human thymocytes except the DN subset and chemotactic activity of these subsets correlated with the expression of CCR5 (Dairaghi et al., 1998). These contrasting results indicate chemokine-mediated migration in relation to thymic differentiation is likely to be an extremely complex series of events to decipher.

Chemokine receptors expressed by more mature thymocyte subsets include CCR3, CCR4 and CCR7. Murine $CD4^+8^+69^+$ thymocytes, a subset that has putatively undergone positive selection, express CCR4 mRNA transiently which is replaced by expression of CCR7 as thymocytes mature to SP cells (Suzuki et al., 1999). Ligands for these two chemokine receptors, including thymus-expressed chemokine, TARC and ELC, are expressed in the medulla suggesting a role in trafficking of more mature thymocytes. Mature $CD8^+$ SP cells express CCR3 and one of its ligands, eotaxin, is expressed in the thymic medulla (Franz-Bacon et al., 1999). However, eotaxin causes chemotaxis of both DP and SP subsets contrary to the lack of detectable expression of CCR3 on DP and $CD4^+$ SP cells. As more is learned about chemokines and their role in thymic migration, it will be of interest to determine how they function in relation to other adhesion and motility receptors.

### RHAMM and HA

RHAMM was originally isolated as a HA-binding protein from locomoting chick heart fibroblasts (Turley, 1989) and subsequent molecular cloning indicated the nucleotide sequence is distinct from the HA binding protein, CD44 (Hardwick et al., 1992). Examination of a number of developing tissues in which cells are required to be migratory indicates RHAMM is expressed and functions as a motility receptor (Pilarski et al., 1999; Gares et al., 1998; Nagy et al., 1995; Kornovski et al., 1994; Pilarski et al., 1993; Boudreau et al., 1991; Turley at al., 1990). RHAMM is also over-expressed by malignant cells isolated from multiple myeloma patients and human breast tumors (Crainie et al., 1999; Wang et al., 1998). Transfection of fibroblasts with RHAMM causes non-tumorigenic cells to become highly tumorigenic which implicates RHAMM as a potent oncogene and emphasizes the importance of this receptor as a mediator of motility (Hall et al., 1995).

Sequence analysis of genomic murine RHAMM indicates the RHAMM gene is comprised of 14 exons and similar to CD44, splice variants are observed (Entwistle et al., 1995). Recently, four additional exons have been identified in the open reading frame of the murine RHAMM gene (Fieber et al., 1999). Three different species of RHAMM transcripts have also been isolated from human B and plasma cells of multiple myeloma patients (Crainie et al., 1999). Two of the three transcripts contain deleted regions corresponding to transcriptional excision of exons, but the functional significance of these variant transcripts is unknown. At the protein level, immunoblots indicate *ex vivo* cells and various cell lines express RHAMM of two or three different apparent molecular weights (Wang et al., 1996; Nagy et al., 1995; Kornovski et al., 1994). As yet, it is unknown whether the variable size of RHAMM is due to translation of splice variants, variation in carbohydrate addition, phosphorylation or a combination of these processes.

Murine and human RHAMM sequences are highly conserved with the majority of sequence variation occurring in the amino terminus (Wang et al., 1996). RHAMM also contains two translation start codons. Translation of human RHAMM mRNA from the +1 position would give rise to a 726 aa protein, whereas translation from the second start codon would yield a protein of 610 aa. Variant usage of these start codons is currently being investigated and could provide another explanation for the size variation of RHAMM proteins.

The HA binding motif was characterized using a RHAMM expression system and consists of two basic amino acids with an intervening span of seven non-acidic amino acids ($BX_7B$) (Yang et al., 1994; 1993). HA binding is optimal if at least one of the intervening residues is also basic. RHAMM contains two HA binding motifs near the carboxy end of the protein that are encoded by exons 12 and 13. HA binding to both domains appears to be additive, thus the two HA binding domains apparently function independent of one another. This HA binding motif has also been identified in the hyaladherins CD44 and link protein, thus may be a universally used HA binding motif.

Consistent with the role of RHAMM as a motility receptor, motile cells express cell surface RHAMM as indicated by FACScan analysis and both fluorescent and confocal microscopy (Pilarski et al., 1999; Crainie et al., 1999; Masellis-Smith, et al., 1996; Nagy et al., 1995; Kornovski et al., 1994; Pilarski et al., 1993). However, there is also substantial expression of an intracellular form of RHAMM (Gares et al., under revision; Pilarski et al., 1999; Assmann et al., 1998; Hofmann et al., 1998; Nagy et al., 1995). Various model systems indicate RHAMM-mediated motility can occur very rapidly (between 1 to 6 H) following wounding or subculture of cell lines (Savani et al., 1995; Turley, 1992). The addition of HA also rapidly upregulates cell motility which can be blocked by RHAMM-specific antibody. This rapid adaptation of cells to a change in their microenvironment suggests HA may modulate RHAMM expression and cell behavior. We have chosen to further explore this interaction using *ex-vivo* human thymocytes. We hypothesized that developing cells such as thymocytes require motility receptors during migratory phases of development. Since a number of differentiating cell types express RHAMM, we reasoned RHAMM might be a likely candidate to mediate motile behavior for thymocytes. We will describe our work to date and put forward a model of how RHAMM interacts with integrins and HA to mediate motility.

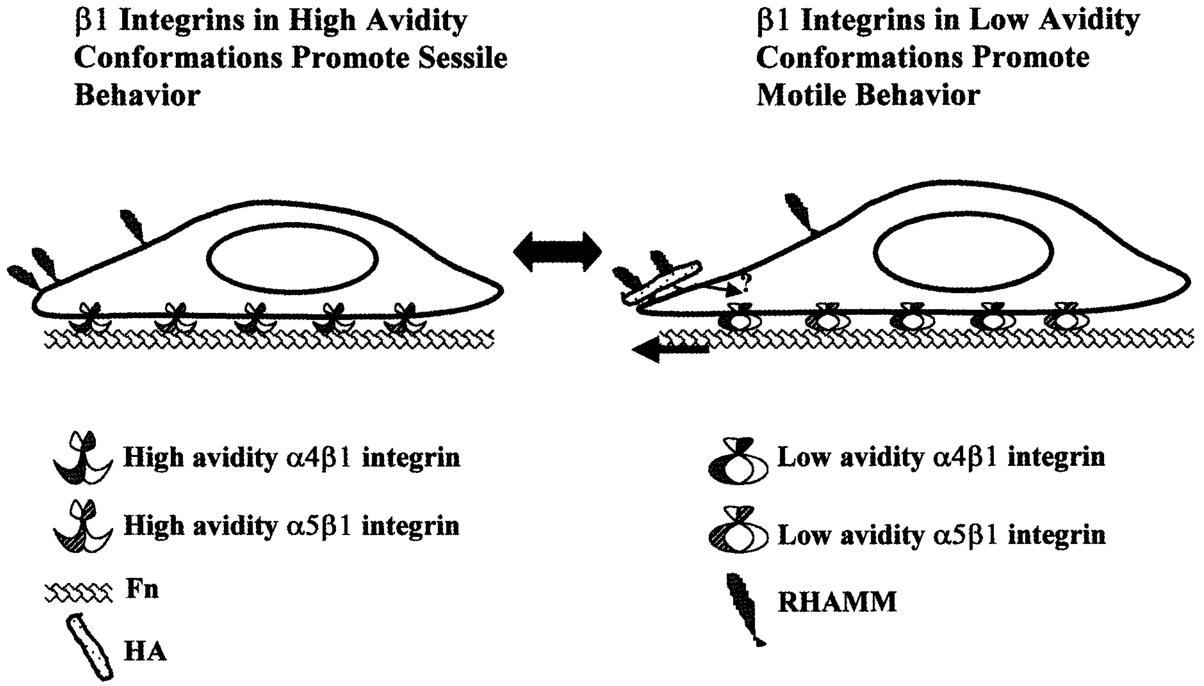

FIGURE 1 The Balance Between Adhesion and Motility is Mediated by Integrin Avidity. When α4β1 and α5β1 are in high avidity binding conformations, human thymocytes adhere strongly to Fn and sessile behavior predominates. HA binding to RHAMM may initiate signals that alter integrins to low avidity binding conformations. Integrins then maintain weakly adhesive contact with Fn, which promotes motile behavior. Thymocytes can revert to either behavior depending on signals received from stromal cells or the microenvironment during differentiation

### A BALANCE EXISTS BETWEEN ADHESION AND MOTILITY

### Interactions Between β1 Integrins And RHAMM

FACScan analysis of unfractionated *ex-vivo* human thymocytes stained with anti-RHAMM monoclonal antibodies (Mabs) indicates RHAMM is expressed at low density by a subset of thymocytes, the majority of which express a moderate to high level of CD3 (Pilarski et al., 1993). To determine if RHAMM expression correlates with developmental subsets of thymocytes based upon expression of CD4 and CD8, three color staining was done. Although MN thymocytes did not express RHAMM, both DP and SP subsets contain $RHAMM^+$ cells. Analysis of the motility of DP cells ($CD4^+8^+$ or $CD3^{med}$) and SP cells ($CD4^+CD8^-$, $CD4^-CD8^+$ or $CD3^{hi}$) using video time-lapse microscopy indicates both DP and $CD4^+$ SP subsets contain motile cells and Mabs to RHAMM significantly inhibit motility as do Mabs to α4, α5 and β1 integrins (Gares et al., 1998). However, Mabs to CD44 have no effect on motility. Although the extent of inhibition by Mabs to integrins or RHAMM is comparable, using video time-lapse microscopy to quantitate motility by observing cellular behavior, we detect a qualitative difference in the blocking activity of the RHAMM Mabs versus the β1 integrin Mabs. Anti-RHAMM Mabs inhibit motility, but cell adherence is unaffected or increased. Function blocking Mabs to the β1 integrin components cause the cells to be non-adherent, suggesting β1 integrins are required by motile thymocytes to make weak adhesive contacts with substrate, providing traction for locomoting cells. Adhesion and motility may be viewed as opposing cellular behaviors in which adhesion receptors are operational while cells remain sessile and are non-functional while cells are motile. However, these observations suggest instead that adhesion receptors remain functional while cells are motile. Strong adhesive contacts must be "broken" to enable a cell to begin to locomote. Therefore, one of the requirements of motility is to decrease strong adhesion. A motile cell still requires contacts with substrate while re-organizing all the cellular machinery required for movement and weak adhesive contacts may be required for motility receptors to function. This implies motile behavior is dependent on the balance between strongly adhesive and weakly adhesive contacts. Thus, increasing the avidity of integrin binding should cause cells to become less motile. To test this possibility, we used a Mab to β1 integrin that, upon binding, forces the integrin into a high avidity binding conformation (Gares et al., 1998). Thymocytes pre-treated with this Mab are less motile and have a concomitant increase in adhesion. This indicates that strong adhesion by integrins is dominant over RHAMM-mediated motility. Forcing β1 integrins into the high avidity binding conformation tips the balance in favor of adhesion. The corollary of this is that to maintain motility, thymocytes must decrease high avidity integrin interactions between stromal elements and ECM. It is possible that RHAMM-mediated signaling may cause integrins to alter to low avidity binding conformations or that separate signals alter integrin function and RHAMM expression maintains this state (Fig. 1). HA binding to RHAMM causes net de-phosphorylation of focal adhesion kinase (FAK) (Hall et al., 1994), which reduces the adhesiveness of focal adhesions, thus one function of RHAMM may be to maintain low avidity integrin binding conformations.

This hypothesis implies a functional relationship exists between integrins and RHAMM. To characterize this relationship experimentally, we examined expression and function of these receptors at an early point in differentiation prior to detectable RHAMM expression (Gares and Pilarski, 1999). MN thymocytes were purified using bead depletion or bead depletion in combination with cell sorting to remove $CD3^{+}4^{+}8^{+}19^{+}$ cells. FACS analysis of these cells showed a lack of RHAMM expression, but moderate expression of α4, α5, α6 and β1 integrin subunits. MN thymocytes were then cultured on uncoated or Fn-coated tissue culture wells over a period of several days and expression of RHAMM and integrin subunits was monitored over time. MN thymocytes cultured *in vitro* for two days, upregulate expression of the β1 integrin subunits and RHAMM in the presence or absence of Fn. This suggests thymocytes undergoing *in vitro* differentiation become migratory and likely use RHAMM to mediate migratory behavior at this point in development. Although Fn is not required to upregulate short term RHAMM expression, thymocytes cultured for longer periods (up to day 5 or 7) maintain RHAMM expression only in the presence of Fn. This implies prolonged RHAMM expression may be dependent upon signals mediated by integrins binding to their ligand. Alternatively, RHAMM may interact directly or indirectly with Fn to maintain expression. To test whether strong adhesive contacts of integrins affect RHAMM expression, MN thymocytes were cultured in Fn-coated wells containing immobilized Mabs to α4, α5, or β1 integrins. Prolonged RHAMM expression is inhibited when Fn-coated wells contain these immobilized Mabs. Control Mabs or anti-RHAMM Mabs do not affect the prolonged expression of RHAMM. Immobilized, function-blocking Mabs to each of these integrin components also decreased RHAMM mRNA expression. This data indicates strong adhesion (emulated by integrin binding to immobilized Mabs) mediated by α4β1 and α5β1 is not only dominant over RHAMM-mediated motility as previously discussed, but also that strong adhesion actually inhibits RHAMM expression. By extrapolation, the alteration of sessile behavior by decreasing integrin avidity must rely on a RHAMM-*in*dependent mechanism, at least at this stage of development. Since chemokine receptors are operational at this stage of development and stromal cells secrete chemokines, these receptors may function to decrease integrin avidity.

Since RHAMM expression is upregulated in the presence or absence of Fn, this suggests MN thymo-

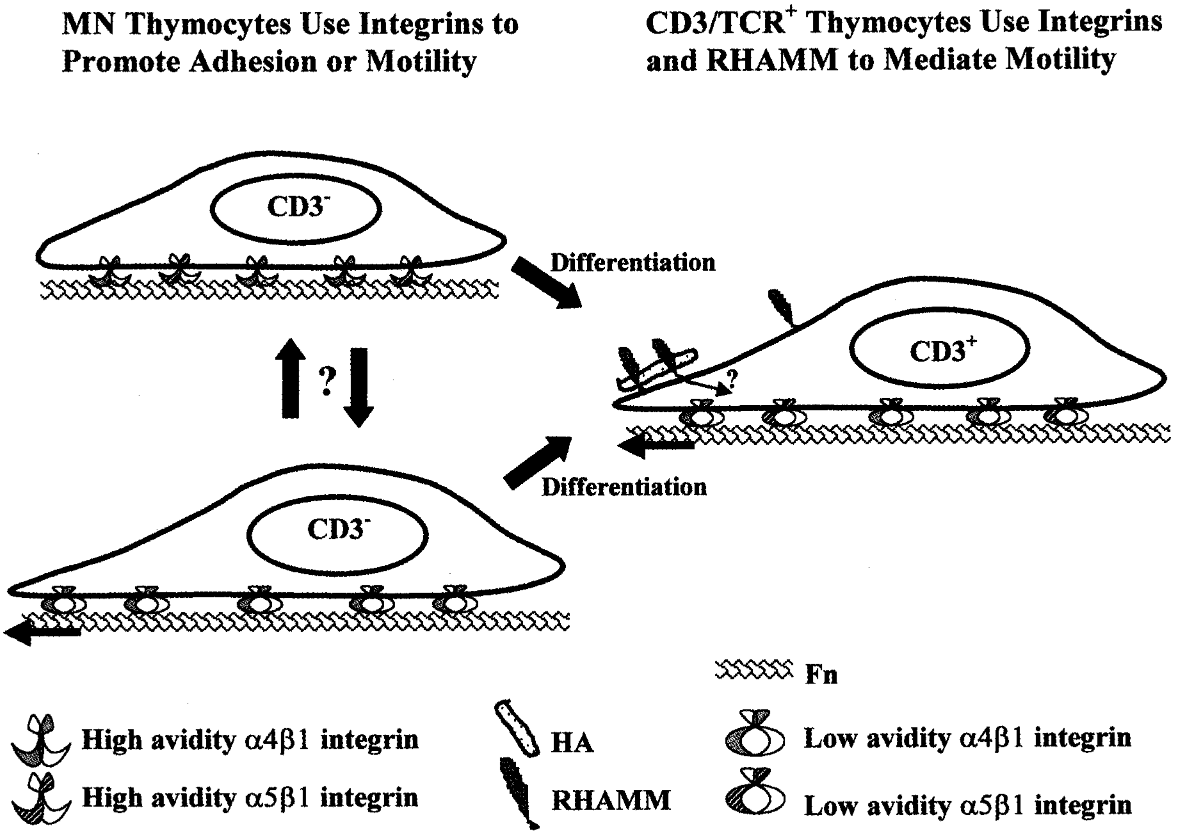

FIGURE 2 Differentiation of MN Thymocytes Correlates With Low Avidity Binding Conformations of α4β1, α5β1 and Onset of RHAMM Expression. High avidity binding conformations of α4β1 and α5β1 integrins maintain sessile behavior for human MN thymocytes by mediating strong binding to the ECM component Fn (as shown) or to stromal cells. α4β1 integrins in low avidity binding conformations mediate motility for MN thymocytes by mediating weak adhesive contacts with ECM components. Highly adhesive MN thymocytes might be at a different developmental stage than motile MN thymocytes, but the sequence in development is unknown at this time. As MN thymocytes differentiate and express $CD3^+$, they begin to express RHAMM. HA binding to RHAMM mediates motility. Integrins are then maintained in low avidity binding conformations to mediate weak adhesive contacts with the ECM and provide traction for locomoting thymocytes cytes receive signals *in vivo* to become migratory. The requirement for Fn to maintain RHAMM expression suggests RHAMM expression and presumably function, is dependent upon integrin interactions with substrate. This interaction is likely via weak adhesive contacts. It also suggests integrins function either directly or indirectly to regulate locomotory behavior by controlling RHAMM expression.

To assess the function of integrins and RHAMM, MN thymocytes and cultured thymocytes were pre-treated with Mabs to integrin subunits or RHAMM followed by analysis of adhesion and motility using video time-lapse microscopy. Using this experimental system we believe both weak and strong adhesion are measured. MN thymocytes contained 31% adherent cells and 40% motile cells. Mabs to β1 and α4 significantly inhibit both adhesion and motility indicating α4β1 is the major mediator of adhesion and motility for undifferentiated thymocytes. Anti-RHAMM Mabs do not inhibit adhesion or motility correlating with the lack of detectable RHAMM expression and verifying motility of MN thymocytes is RHAMM *in*dependent. Using a fluorescence-based adherence assay in which thymocytes adhere and are

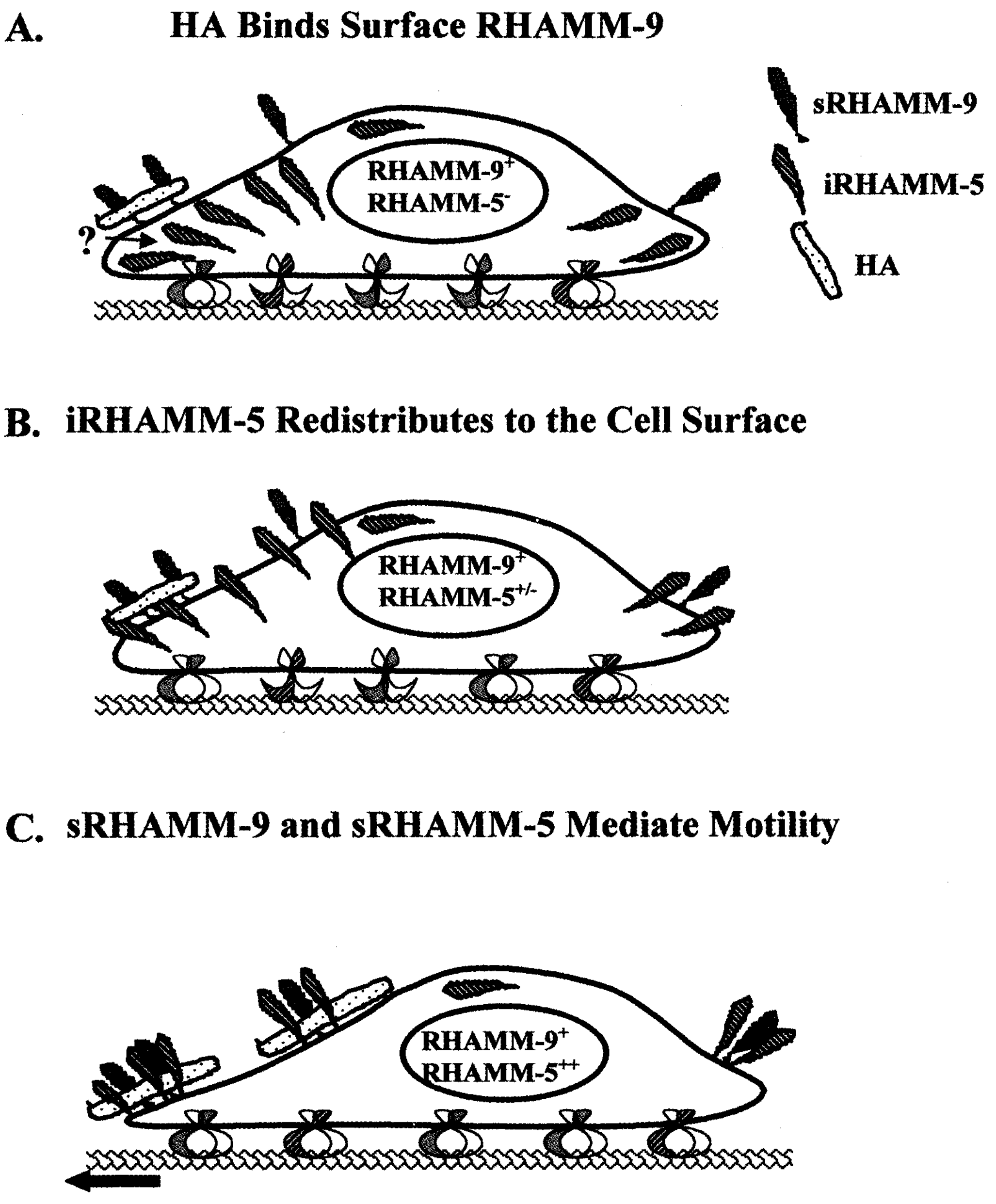

FIGURE 3 HA Binding Redistributes iRHAMM-5 to the Cell Surface Where It Becomes A Participant in Motility. Thymocytes express a low density of sRHAMM-9 and a higher density of iRHAMM-5. Prior to HA binding to sRHAMM-9, integrins may bind Fn with high avidity and maintain sessile behavior for thymocytes (A). HA binding to sRHAMM-9 initiates signals that result in a redistribution of iRHAMM-5 to the cell surface and conversion of integrins to low avidity binding conformations (B). At the cell surface, sRHAMM-5 associates with sRHAMM-9 to form HA binding complexes that mediate motility. Low avidity binding conformations of integrins mediate weak adhesive contacts with Fn that provide traction for the locomoting thymocytes (C)

then subjected to strong shear forces produced by pipetting, we found Mabs to α4 and β1 also inhibit strong adhesion, but in contrast to the results of the video time-lapse analysis, Mabs to α5 also inhibit adherence. We have interpreted this to mean that binding to α5 by this particular Mab does not interrupt adhesive contacts with ligand when shear forces are low. When an adhesion assay is used that incorporates strong shear forces to remove non-adherent and weakly adherent cells, Mabs to α5 inhibit adhesion indicating this epitope is required to make contact with ligand to maintain strong adhesion by α5β1. Therefore, MN thymocytes use α5β1 to maintain strong adhesion in combination with α4β1. α4β1 is also used by MN thymocytes to mediate motility. Alternatively, α4β1 may mediate motility in conjunction with an unidentified motility receptor.

We also assessed the motility of differentiated MN thymocytes by culturing MN cells on Fn-coated plates for two days. At this stage of thymocyte development, motility is now inhibited by anti-RHAMM Mabs. By day 2 of culture, thymocytes also express CD3/TCR, indicating that differentiation of thymocytes correlates with RHAMM expression and function. These experiments may reflect how low and high avidity integrin interactions operate in the thymus at early stages of differentiation (Fig. 2). In terms of thymocyte development, interaction of integrins with ligands expressed by stromal elements or ECM may regulate cell behavior by altering integrin conformations. Integrins on thymocytes may be held in high avidity conformations during stromal interactions to prolong contact such that thymocytes receive selection signals or growth factors. Integrins may be in a low avidity binding conformation when in contact with ECM to promote migration in cell-free areas of the thymus. Cortical epithelial cells express VCAM and it has been proposed interactions between α4β1 on thymocytes and VCAM on stromal cells is a high avidity interaction associated with sessile behavior by thymocytes as they undergo positive selection. Interaction of α4β1 with Fn is a lower avidity interaction likely to support migration (Salomon, et al., 1997). The immobilized anti-integrin Mabs may mimic the high avidity interactions of thymocytes with stromal elements that function to "suppress" the initiation of motile behavior by inhibiting RHAMM expression. MN thymocytes likely remain sessile as they rearrange TCR genes, but TCRβ expression leads to a burst of proliferation, which presumably requires a decrease in high avidity interactions of proliferating cells with stromal cells. RHAMM expression may be initially upregulated at this point in development, then maintained by low avidity interactions of integrins with Fn. Once RHAMM is expressed, HA binding with RHAMM might also function to maintain low avidity integrin binding, since HA binding to RHAMM causes a net dephosphorylation of FAK. However the initial decrease in integrin avidity occurs prior to RHAMM expression, since α4β1 integrin mediates motility, perhaps with another unidentified receptor, prior to RHAMM expression.

### Interaction Of RHAMM With HA

The ability of integrins to shift between low and high avidity binding conformations enables a cell to rapidly alter cellular behavior. If RHAMM function is related to integrin function, RHAMM may also be expressed in a manner that enables rapid adaptation to microenvironmental cues. To study this we have examined the expression and function of RHAMM in response to HA.

We used two different anti-RHAMM Mabs, 3T3–5 and 3T3–9, to stain thymocytes (Gares et al., submitted). The epitope of RHAMM detected by Mab 3T3–9 is expressed at low density on the cell surface of 30 to 60% of thymocytes. When thymocytes are stained sequentially with Mab 3T3–9 then HA conjugated to FITC (HA-FITC) and examined by confocal microscopy, there is co-localization of RHAMM expression at sites of HA-FITC binding. CD44 is expressed by human thymocytes, but HA binding does not correlate strongly with sites of CD44 expression. This suggests RHAMM is the predominant cell surface HA binding receptor used by human thymocytes.

A second anti-RHAMM Mab, 3T3–5, demonstrates very low to negligible surface binding to thymocytes. However, both anti-RHAMM Mabs bind almost equivalently to a RHAMM fusion protein as assessed by an ELISA. Cell surface RHAMM may lack or conceal the epitope of Mab 3T3–5. Alternatively, these two Mabs may recognize RHAMM epitopes expressed by alternate isoforms of RHAMM. Several reports indicate cells express an intracellular form of RHAMM (Pilarski et al., 1999; Hofmann et al., 1998; Assmann et al., 1998; Nagy et al., 1995). To determine if thymocytes express intracellular RHAMM, cells were permeabilized, then stained with anti-RHAMM Mabs. FACScan analysis and confocal microscopy indicate Mab 3T3–5 binds an intracellular form of RHAMM expressed by thymocytes that we have arbitrarily named **iRHAMM-5** (intracellular RHAMM bound by Mab 3T3-**5**). Approximately 70% of thymocytes express iRHAMM-5 and all iRHAMM-$5^+$ cells are $CD3^+$. Mab 3T3–9, which binds cell surface RHAMM and which we have named **sRHAMM-9** (cell surface RHAMM bound by Mab 3T3-**9**) did not stain permeabilized thymocytes suggesting this epitope is destroyed by permeabilization. Analysis of the predicted amino acid sequence of human RHAMM by various motif search programs predict RHAMM is an intracellular protein. The amino acid sequence contains no obvious signal sequence or transmembrane domain, although there is abundant evidence that RHAMM is expressed at the cell surface of normal and transformed cells (Pilarski et al., 1999; Crainie et al., 1999; Dowthwaite et al., 1998; Teder et al., 1995; Masellis-Smith et al., 1996; Nagy et al., 1995; Pilarski et al., 1993). Rat and human carcinoma cell lines express iRHAMM and the groups involved with this work have speculated iRHAMM functions as an intracellular HA binding receptor (Assmann et al., 1998; Hofmann et al., 1998). Permeabilized thymocytes stained sequentially with Mab 3T3–5 then HA-FITC and examined by confocal microscopy show a co-localization of HA binding with sites of iRHAMM-5, suggesting that in thymocytes, iRHAMM-5 may function in this capacity. Endocytosed HA mediates numerous cellular functions and iRHAMM-5 may participate in intracellular trafficking of HA. However, RHAMM is also clearly expressed on the cell surface of thymocytes and many other cells, but the mechanism of surface expression is still unknown. RHAMM has previously been reported to exist in a multimer called HARC (HA receptor complex) (Turley, 1992). The identity of all components of this complex is unknown, but interaction of RHAMM with other membrane or transmembrane proteins may enable surface expression of RHAMM. Alternatively, sRHAMM may be expressed as a gpi-anchored protein.

Preliminary experiments were done to determine if HA binding affects surface expression of RHAMM. Intact thymocytes were pre-treated with soluble HA-FITC then stained with Mabs to RHAMM. Staining patterns of Mab 3T3–9 are indistinguishable between HA-treated and untreated thymocytes indicating HA does not block the binding site of this Mab nor does HA detectably affect expression of sRHAMM-9. Surprisingly, HA pre-treatment resulted in detection of cell surface RHAMM by Mab 3T3–5. Two explanations seem possible. HA binding may cause a conformational change of sRHAMM-9 revealing the epitope of Mab 3T3–5 without altering the epitope of Mab 3T3–9. Alternatively, thymocytes may express two isoforms of RHAMM that function differently in response to HA binding. Mab 3T3–5 stains a greater number of HA-treated thymocytes with a greater intensity of staining than can be accounted for by the low density of sRHAMM-9 expression. This indicates HA binding increases the number of $RHAMM^+$ cells and increases the density of RHAMM expressed at the cell surface as detected by Mab 3T3–5. This might be achieved by a redistribution of iRHAMM-5 to the cell surface. As depicted by Fig. 3A, thymocytes express a low density of sRHAMM-9 at the cell surface and an intermediate density of intracellular iRHAMM-5. Thus, staining of intact thymocytes with anti-RHAMM Mabs indicates that, on the cell surface, thymocytes are RHAMM-$9^+$ and RHAMM-$5^-$. HA binding to sRHAMM-9 generates signals that cause redistribution of iRHAMM-5 to the cell surface (now sRHAMM-5) enabling binding by Mab 3T3–5 (Fig. 3B and 3C) such that the surface phenotype of HA-treated thymocytes is RHAMM-$9^+$ and RHAMM-$5^+$. Consistent with this model, a large proportion of thymocytes express iRHAMM-5 and a redistribution of iRHAMM-5 to the cell surface would account for the increased number of sRHAMM$^+$ cells and the overall increase of receptor density at the cell surface.

This model proposes that thymocytes maintain an intracellular store of RHAMM that is unable to mediate motility unless redistributed to the cell surface by appropriate microenvironmental cues. Potentially, this would enable cells to rapidly alter from sessile to motile behavior presumably in response to signals received during differentiation. A prediction of this model is that HA should be required to mediate motility and iRHAMM-5 should mediate motility when redistributed to the cell surface. Surprisingly, the number of motile thymocytes is approximately 18% on Fn-coated wells in the presence *or* absence of HA. Using wells coated only with HA, motility is almost negligible, again indicating thymocytes require contact with Fn to mediate motility. To understand how thymocytes mediate RHAMM-mediated motility in the absence of exogenous ligand, we hypothesized that thymocytes may synthesize their own HA. To test this hypothesis, thymocytes were pre-treated with hyaluronidase to remove endogenous HA and motility was assessed on Fn-coated wells in the absence or presence of exogenous HA (Gares et al., 1998). Motility is ablated when thymocytes are pre-treated with HAase, but addition of exogenous HA restores motile behavior. This indicates HA is required to mediate random motility for thymocytes and that thymocytes synthesize their own HA. Hyaluronan synthase (HAS) synthesizes HA at the inner surface of the plasma membrane and extrudes the polysaccharide externally during synthesis. Three mammalian HAS genes have been identified (Spicer et al., 1997; 1996; Itano & Kimata, 1996; Watanabe & Yamaguchi, 1996; Klewes et al., 1993) and we have found human thymocytes express mRNA for at least one of these enzymes. We are currently examining the expression of HAS among different thymocyte subsets to determine if HAS expression correlates with differentiation of the thymocytes. The observation that thymocytes have the potential to synthesize their own HA is provocative and suggests differentiating thymocytes do not just respond to their microenvironment, but participate in creating a microenvironment likely to promote RHAMM-mediated motility. It also predicts RHAMM is likely to be in close physical proximity to HAS. Thus, once RHAMM and HAS are expressed, they may function in conjunction with integrins to push the behavioral balance toward migration (Fig. 1).

To determine if both sRHAMM-9 and sRHAMM-5 participate in motility, thymocytes were pre-treated with anti-RHAMM Mabs, washed and motility was assessed on Fn-coated wells in the presence or absence of HA (Gares et al., submitted). Mab 3T3–9 significantly inhibits motility in the presence or absence of HA indicating sRHAMM-9 is a functional mediator of motility. If HA binding to sRHAMM-9 is required, endogenous HA must be synthesized in sufficient quantity to function in mediating motility. This again implies HAS may be in close physical proximity to sRHAMM. In contrast, Mab 3T3–5 decreases motility marginally in the absence of exogenous HA, but inhibits motility almost completely in the presence of HA. This corresponds to the expression data indicating redistribution of iRHAMM-5 to the cell surface requires an exogenous source of HA. The redistribution of iRHAMM-5 must require high levels of HA and in our experimental system, this can only be achieved when exogenous HA is added. Once at the cell surface, sRHAMM-5 participates in motility. These experiments indicate both sRHAMM-9 and sRHAMM-5 mediate motility and both forms of RHAMM are required for motility, since Mabs to either form of RHAMM significantly decrease motility. Mab binding does not block HA binding to RHAMM, therefore the inhibition of motility by Mab binding interferes with other functions of RHAMM. Binding patterns of the two anti-RHAMM Mabs suggest thymocytes express two functionally distinct isoforms of RHAMM. PCR analysis also indicates thymocytes express transcripts for two isoforms of RHAMM and additional work is underway to determine whether these transcripts translate into proteins with the properties we have described.

### Do Thymocytes Express Two RHAMM Isoforms?

sRHAMM-9 and iRHAMM-5 localize to the cell surface and an intracellular site, respectively, in untreated *ex-vivo* thymocytes, implying thymocytes express two isoforms of RHAMM and each performs different functions for cells. The staining patterns by the two Mabs are also consistent with the expression of two RHAMM isoforms, since staining patterns indicate the two forms of RHAMM express different epitopes. HA pre-treatment of thymocytes enables Mab 3T3–5 to bind cell surface RHAMM which is consistent with a different, intracellular isoform of RHAMM being redistributed to the cell surface. In addition, HA pre-treatment does not increase surface staining by Mab 3T3–9. Binding by HA is not expected to affect either Mab binding to their respective epitopes, since confocal analysis indicates both RHAMM isoforms can be dual stained with Mab 3T3–5 or 3T3–9 and HA-FITC.

However, sRHAMM-9 and iRHAMM-5 could be genetically identical, but occur in both cellular compartments as a consequence of endocytosis of HA. RHAMM-mediated endocytosis of HA has been reported (Collis et al., '1998; Masellis-Smith et al., 1996) and internalized sRHAMM-9 could contribute to the intracellular pool of RHAMM that we have identified as iRHAMM-5. Receptor recycling could also account for the presence of RHAMM in both cellular compartments. We have tested the possibility that sRHAMM is a gpi-anchored protein. Thymocytes were pre-treated with phosphoinositol-specific phospholipase C (PLC) followed by analysis of motility on Fn-coated wells in the absence or presence of exogenous HA (Gares et al., submitted). Motility of PLC-treated thymocytes is ablated if behavior is assessed in the absence of exogenous HA. However, if exogenous HA is present, motility is restored and PLC-treated cells contain the same number of motile cells as untreated controls. The ability of PLC treatment to inhibit RHAMM-mediated motility suggests thymocytes express RHAMM as a gpi-anchored protein. Consistent with our model, motility is restored to PLC-treated thymocytes if exogenous HA is added because iRHAMM-5 is redistributed to the cell surface. However this could also be consistent with expression of only one isoform of RHAMM. If sRHAMM is a gpi-anchored protein, it might cycle from the cell surface to intracellular sites in lipid rafts in response to HA binding and contribute to the intracellular pool of RHAMM until it cycles back to the cell surface.

Mab staining patterns indicate sRHAMM-9 and iRHAMM-5 express different epitopes. However, one form of RHAMM could contain both epitopes, but Mab binding might be dependent on the conformation RHAMM assumes in different cellular locations. For example, at the cell surface, RHAMM might associate with the components of HARC and assume a conformation hiding the 3T3–5 binding epitope while preserving the 3T3–9 epitope. If this occurs, it would result in negligible binding to cell surface RHAMM by Mab 3T3–5 giving the appearance that sRHAMM-9 and iRHAMM-5 are different isoforms because they are specifically recognized by different Mabs. Evidence against this possibility is that Mab 3T3–5 continues to bind to iRHAMM-5 after it is redistributed to the cell surface where it should assume the `cell surface' conformation. It is plausible that sRHAMM-5 may interact with alternative cell surface proteins than does sRHAMM-9 when it is initially redistributed to the cell surface. This would maintain RHAMM-5, at least transiently, in a conformation that reveals the 3T3–5 epitope and hides the 3T3–9 epitope. Definitive evidence to determine whether the two Mabs bind two distinct isoforms of RHAMM requires expression of the previously described RHAMM mRNA isoforms into an expression vector followed by analysis of the expressed proteins.

### How Do sRHAMM-9 And iRHAMM-5 Function To Mediate Motility?

Although Mab binding profiles provide suggestive but inconclusive evidence for the existence of two distinct RHAMM isoforms in human thymocytes, our motility analyses indicate sRHAMM-9 and iRHAMM-5 function differently, supporting our hypothesis that thymocytes express two RHAMM

isoforms. Thymocytes treated with HAase are not motile which indicates HA is absolutely required by thymocytes to mediate motility. HAase-treated thymocytes have the phenotype sRHAMM-9$^+$/sRHAMM-5$^-$, thus RHAMM surface expression is not affected (unpublished observations). The requirement for HA may indicate HA binding to sRHAMM-9 mediates motility, generates signals or redistributes iRHAMM-5 to the cell surface. Redistributed sRHAMM-5 then mediates motility alone or in combination with sRHAMM-9. Mab 3T3–9 inhibits motility in this assay system, but we do not know if motility is inhibited directly or whether upstream events are inhibited by Mabs binding to sRHAMM-9. Anti-RHAMM Mabs do not block HA binding, but may block interaction of sRHAMM-9 with other proteins required to generate signals or mediate motility. HA binding to sRHAMM results in net de-phosphorylation of FAK (Hall et al., 1995), thus HA binding may mediate signals through sRHAMM-9 and perhaps other components of HARC. This signaling cascade may initiate integrins to adopt, or maintain low avidity binding conformations required to promote motile behavior. If Mab 3T3–9 inhibits signaling by sRHAMM-9, integrins will be maintained in a high avidity conformation and adhesion becomes the dominant behavior.

In contrast to Mab 3T3–9, Mab 3T3–5 blocks RHAMM-mediated motility only in the presence of HA. In the absence of exogenous HA, iRHAMM-5 remains inside the cell and Mab 3T3–5 cannot bind, thus cannot inhibit motility. Synthesis of endogenous HA during the period of Mab pre-treatment must be insufficient to mobilize iRHAMM-5 to the cell surface in sufficient quantity for Mabs to bind and cause subsequent inhibition of motility. Most importantly, this data indicates the absolute requirement for RHAMM-5 to mediate motility. By extrapolation, motile behavior requires redistribution of iRHAMM-5 to the cell surface where it can participate in mediating motility. Interestingly, although PLC treatment inhibits thymocyte motility, FACS analysis of PLC-treated thymocytes indicates only a partial decrease of staining by Mab 3T3–9. This indicates sRHAMM-9 is not completely removed from the cell surface by PLC and suggests sRHAMM-9 may not be a gpi-anchored protein. Alternatively, intermolecular interactions of sRHAMM-9 with HA or other surface proteins may protect the gpi anchor from cleavage by PLC. An alternative possibility is that PLC treatment inhibits motility by cleaving sRHAMM-5. Thymocytes stained with Mab 3T3–5 demonstrate very weak staining profiles and this indicates very low to no density of receptor at the cell surface. However, PLC-treated thymocytes subsequently stained with Mab 3T3–5 demonstrate even weaker staining profiles in comparison to untreated cells. Although Mab 3T3–5 demonstrates negligible staining of *ex vivo* thymocytes, sRHAMM-5 may be present at very low density on most thymocytes at any given time. iRHAMM-5 may constantly cycle from an intracellular compartment to the cell surface perhaps in response to low levels of endogenous HA. At the cell surface it appears to be expressed as a gpi-anchored protein or associates with a gpi-anchored protein, thus is susceptible to cleavage by PLC.

The ability of both Mabs to block motility indicates both RHAMM isoforms are functional mediators of motility. This suggests sRHAMM-9 and sRHAMM-5 may combine to form multimers at the cell surface and these multimers are the functional mediators of motility (Fig. 3C). There is indirect evidence suggesting RHAMM receptors associate in multimeric complexes to mediate motility. A RHAMM gene truncated to remove the HA binding domains behaves as a dominant suppressor of RHAMM function when transfected into tumorigenic cells (Hall et al., 1995). The transfected cells become less motile and form large stable focal adhesions indicating RHAMM function is downregulated. These cells express wild type RHAMM as well as mutant RHAMM, yet mutant RHAMM dominates in mediating cellular behavior. This suggests wild type RHAMM and mutant RHAMM are unable to form multimers or form multimers that are unable to function normally because they lack the full complement of HA binding domains. Consistent with the idea that RHAMM only mediates motility when it forms a multimeric complex, Mab binding to either isoform of RHAMM

might interfere with the ability of sRHAMM-9 to associate with sRHAMM-5. Redistributed sRHAMM-5 may combine with sRHAMM-9 in HARC. Alternatively, sRHAMM-9 may dissociate from this complex and combine with redistributed sRHAMM-5 to mediate motility. This latter possibility implies sRHAMM-9 may be involved in generating signals when in association with other components of HARC and mediates motility when in association with sRHAMM-5. The high density of sRHAMM-5 following HA treatment predicts RHAMM complexes are likely to contain two or more sRHAMM-5 receptors interacting with one sRHAMM-9 receptor. Expression of RHAMM in a multimeric complex is likely to increase ligand binding avidity since HA is a relatively linear, polymeric molecule. Only multimers of the basic HA dimer mediate motility, therefore, a multimeric association of RHAMM-5 and RHAMM-9 may be required to bind HA and mediate motility.

### CLOSING REMARKS

This model indicates RHAMM functions to provide thymocytes with a mechanism to rapidly alter from sessile to motile behavior in the presence of appropriate stimuli. The model also predicts that once thymocytes express RHAMM, they may also express HAS in order to maintain endogenous synthesis of HA. Endogenous HA might be required to maintain very low levels of surface RHAMM while cells are sessile. An important postulate of this model is that HA binding by RHAMM either causes integrins to adopt, or maintains integrins in, a low avidity binding conformation which keeps cells in a "ready-state" for migration. Although integrins may control the balance between adhesive and motile behavior before RHAMM is expressed, it seems reasonable that RHAMM should play a role in this decision once it is expressed. Just as there is a balance between negative and positive selection based on TCR:MHC avidity, there is likely to be a balance between sessile and motile behavior based on integrin avidity.

Thymocytes are required to make many "stops and starts" as they differentiate because they must interact with many cells within a given compartment and also must move between compartments during maturation. This model presents a way for cells to rapidly change from sessile behavior to motile behavior and vice versa, thus should fulfill the behavioral requirements of differentiating thymocytes.

### *References*

Amsen, D. and Kruisbeek, A. M. (1998) Thymocyte selection: not by TCR alone. Immunol. Rev. 165:209–229.

Anderson, G., Moore, N. C., Owen, J.J. and Jenkinson, E. J. (1996) Cellular interactions in thymocyte development. Ann. Rev. Immunol. 14:73–99.

Assmann, V., Marshall, J. F., Fieber, C., Hofmann, M. and Hart, I.R. (1998) The human hyaluronan receptor RHAMM is expressed as intracellular protein in breast cancer cells. J. Cell Science. 111:1685–1694.

Berrih, S., Savino, W. and Cohen, S. (1985) Extracellular matrix of the human thymus: immunofluorescence studies on frozen sections and cultured epithelial cells. J. Histochem. Cytochem. 33:655–664.

Borland, G., Ross, J.A. and Guy, K., (1998) Forms and function of CD44. Immunol. 93:139–148.

Boudreau, N., Turley, E. A. and Rabinovitch, M., (1991) Fibronectin, hyaluronan and hyaluronan binding protein contribute to increased ductis arteriosis smooth muscle cell migration. Dev. Biol. 143:235–247.

Boyd, R. L., Tucek, C. L., Godfrey, D. I., Izon, D. J., Wilson, T. J., Davidson, N. J., Bean, A. G. D., Ladyman, H. M., Ritter, M. A. and Hugo, P. (1993) The thymic microenvironment. Immunol. Today 14:445–459.

Boyd, R.L. and Hugo, P. (1991) Towards an integrated view of thymopoiesis. Immunol. Today 12:71–78.

Chan, S., Correia-Neves, M., Benoist, C. and Mathis, D. (1998) CD4/CD8 lineage commitment: matching fate with competence. Immunol. Rev. 165:195–207.

Chang, A. C., Wadsworth, S. and Coligan, J. E. (1993) Expression of merosin in the thymus and its interaction with thymocytes. J. Immunol. 151:1789–1801.

Collis, L., Hall, C., Lange, L., Ziebell, M., Prestwich, R. and Turley, E. (1998) Rapid hyaluronan uptake is associated with enhanced motility: implications for an intracellular mode of action. FEBS Letters 440:444–449.

Crainie, M., Belch, A. R., Mant, M. and Pilarski, L. M. (1999) Overexpression of the hyaluronan receptor RHAMM characterizes the malignant clone in multiple myeloma: identification of three distinct isoforms. Blood 93:1684–1696.

Crisa, L., Cirulli, V., Ellisman, M. H., Ishii, J. K., Elices, M. J. and Salomon, D. R. (1996) Cell adhesion and migration are regulated at distinct stages of thymic T cell development: The roles of fibronectin, VLA4 and VLA5. J. Exp. Med. 184:215–228.

Dairaghi, D. J., Franz-Bacon, K., Callas, E., Cupp, J., Schall, T. J., Tamraz, S. A., Boehme, S., Taylor, N. and Bacon, K. B. (1998) Macrophage inflammatory protein-1$\beta$ induces migration and activation of human thymocytes. Blood 91:2905–2913.

Dardenne, M. and Savino, W. (1994) Control of thymus physiology by peptidic hormones and neuropeptides. Immunol. Today 15:518–523.

Dowthwaite, G. P., Edwards, C. W. and Pitsillides, A. A. (1998) An essential role for the interaction between hyaluronan and hyaluronan binding proteins during joint development. J. Histochem. Cytochem. 46:641–651.

Ellmeier, W., Sawada, S. and Littman, D. (1999) The regulation of CD4 and CD8 coreceptor gene expression during T cell development. Annu. Rev. Immunol. 17:523–554.

Entwistle, J, Hall, C. L. and Turley, E. A. (1996) HA receptors: regulators of signaling to the cytoskeleton. J. Cell. Biochem. 61:560–577.

Entwistle, J., Zhang, S., Yang, B., Wong, C., Li, Q., Hall, C. L., A, J., Mowat, M., Greenberg, H. and Turley, E. A. (1995) Characterization of the murine gene encoding the hyaluronan receptor RHAMM. Gene 163:233–238.

Fehling, H. J. and von Boehmer, H., (1997) Early αβ T cell development in the thymus of normal and genetically altered mice. Curr. Opin. Immunol. 9:263–275.

Fieber, C., Plug, R., Sleeman, J., Dall, P., Ponta, H. and Hofmann, M. (1998) Characterization of the murine gene encoding the intracellular hyaluronan receptor IHABP (RHAMM). Gene. 226:41–50.

Franz-Bacon, K., Dairaghi, D. J., Boehme, S. A., Sullivan, S. K., Schall, T. J., Conlon, P.J., Taylor, N, and Bacon, K. B. (1999) Human thymocytes express CCR-3 and are activated by eotaxin. Blood 10:3233–3240.

Fraser, J. R. E., Laurent, T. C. and Laurent, U. B. G. (1997) Hyaluronan: its nature, distribution, functions and turnover. J. Internal. Med. 242:27–33.

Gares, S. L., Giannakopoulos, N., MacNeil, D., Faull, R. J. and Pilarski, L. M. (1998) During human thymic development, β1 integrins regulate adhesion, motility and the outcome of RHAMM/hyaluronan'engagement. J. Leuk. Biol. 64:781–790.

Gares, S. L., Crainie, M. and Pilarski, L. M. Cellular redistribution of the hyaluronan (HA) receptor RHAMM in human thymocytes is regulated by HA binding. Submitted.

Gares, S. L. and Pilarski, L. M. (1999) β1 integrins control spontaneous adhesion and motility of human progenitor thymocytes and regulate differentiation-dependent expression of RHAMM. Scand J. Immunol. 50:626–634.

Hall, C. L., Yang, B., Yang, X., Zhang, S., Turley, M., Samuel, S., Lange, L. A., Wang, C., Curpen, G. D., Savani, R. C., Greenberg, A. H. and Turley, E. A. (1995) Overexpression of the hyaluronan receptor RHAMM is transforming and is required for H-*ras* transformation. Cell 82:19–28.

Hall, C. L., Wang, C., Lange, L. A. and Turley, E. A. (1994) Hyaluronan and the hyaluronan receptor RHAMM promote focal adhesion turnover and transient tyrosine kinase activity. J. Cell Biol. 126:575–588.

Halvorson, M. J., Magner, W. and Coligan, J. E. (1998) α4 and α5 integrins costimulate the CD3-dependent proliferation of fetal thymocytes. Cell. Immunol. 189:1–9.

Hardwick, C., Hoare, K., Owens, R., Hohn, H. P., Hook, M., Moore, D., Cripps, V., Austen, L., Nance, D. M. and Turley, E. A. (1992) Molecular cloning of a novel hyaluronan receptor that mediates tumor cell motility. J. Cell Biol. 117:1343–1350.

Haynes, B. F. (1986) The role of the thymic microenvironment in promotion of early stages of human T cell maturation. Clin. Res. 34:422–431.

Haynes, B. F. (1984) The human thymic microenvironment. Adv. Immunol. 36:87–141.

Hedrick, S. M and Sharp, L. L. (1998) T cell fate. Immunol. Rev. 165:95–110.

Hofmann, M., Fieber, C., Assmann, V., Gottlicher, M., Sleeman, J., Plug, R., Howells, N., von Stein, O., Ponta, H. and Herrlich, P. (1998) Identification of IHABP, a 95 kda hyaluronate binding protein. J. Cell Sci. 111:1673–1684.

Itano, N. and Kimata, K. (1996) Molecular cloning of human hyaluronan synthase. Biochem. Biophys. Res. Commun. 222:816–820.

Kornovski, B. S., McCoshen, J., Kredentser, J. and Turley, E. A. (1994) The regulation of sperm motility by a novel hyaluronan receptor. Fert. Steril. 61:935–940.

Killeen, N., Irving, B. A., Pippig, S. and Zingler, K. (1998) Signaling checkpoints during the development of T lymphocytes. Curr. Opin. Immunol. 10:360–367.

Knudson, C. B. and Knudson, W. (1993) Hyaluronan-binding proteins in development, tissue homeostasis and disease. FASEB J. 7:1233–1241.

Lagrota-Cândido, J. M., Villa-Verde, D. M. S., Vanderlei Jr., F. H. V and Savino, W. (1996) Extracellular matrix components of the mouse thymus microenvironment. V. Interferon-γ modulates thymic epithelial cell/thymocyte interactions via extracellular matrix ligands and receptors. Cell. Immunol. 170:235–244.

Marrack, P. and Kappler, J. (1997) Positive selection of thymocytes bearing αβ T cell receptors. Curr. Opin. Immunol. 9:250–255.

Masellis-Smith, A., Belch, A. R., Mant, M. J., Turley, E. A. and Pilarski, L. M. (1996) Hyaluronan-dependent motility of B cells and leukemic plasma cells in blood, but not of bone marrow plasma cells, in multiple myeloma: alternate use of receptor for hyaluronan-mediated motility (RHAMM) and CD44. Blood 87:1891–1899.

Nagy, J. I., Hacking, J., Frankenstein, U. N. and Turley, E. A. (1995) Requirement of the hyaluronan receptor RHAMM in neurite extension and motility as demonstrated in primary neurons and neuronal cell lines. J. Neurosci. 15:241–252.

Nikolic-Zugic, J. (1991) Phenotypic and functional stages in the intrathymic development of αβ T cells. Immunol. Today 12:65–70.

Pilarski, L. M., Miszta, H. and Turley, E. A. (1993) Regulated expression of a receptor for hyaluronan-mediated motility on human thymocytes and T cells. J. Immunol 150:4292–4302.

Pilarski, L. M., Pruski, E., Wizniak, J., Paine, D., Seeberger, K., Mant, M. J., Brown, C. B. and Belch, A. R. (1999) Potential role for hyaluronan and the hyaluronan receptor RHAMM in mobilization and trafficking of hematopoietic progenitor cells. Blood 93:2918–2927.

Pilarski, L. M. (1993) Adhesive interactions in thymic development: Does selective expression of CD45 isoforms promote stage-specific microclustering in the assembly of functional adhesive complexes in differentiating T lineage lymphocytes? Immunol Cell Biol 71:59–69.

Ritter, M. A. and Boyd, R. L. (1993) Development in the thymus: it takes two to tango. Immunol. Today. 14:462–469.

Rodewald, H. R. and Fehling, H. J. (1998) Molecular and cellular events in early thymocyte development. Adv. Immunol. 69:1–112.

Salomon, D. R., Crisa, L., Mojcik, C. F., Ishii, J. K., Klier, G. and Shevach, E. M. (1997) Vascular cell adhesion molecule-1 is expressed by cortical thymic epithelial cells and mediates thymocytes adhesion, implications for the function of a4bl (VLA4) integrin in T cell development. Blood 89:2461–2471.

Salomon, D. R., Mojcik, C. F., Chang, A. C., Wadsworth, S., Adams, D. H., Coligan, J. E. and Shevach, E. M. (1994) Constitutive activation of integrin a4b1 defines a unique stage of human thymocyte development. J. Exp. Med. 179:1573–1584.

Savani, R. C., Wang, C., Yang, B., Zhang, S., Kinsella, M. G., Wight, T. N., Stern, R., Nance, D. M. and Turley, E. A. (1995) Migration of bovine aortic smooth muscle cells after wounding injury: the role of hyaluronan and RHAM. J. Clin. Invest. 95:1158–1168.

Savino, W., Villa-Verde, D. M. S. and Lannes-Vieira, J. (1993) Extracellular matrix proteins in intrathymic T-cell migration and differentiation? Immunol. Today 14:158–161.

Sawada, M., Nagamine, J., Takeda, K., Utsumi, K., Kosugi, A., Tatsumi, Y., Hamaoka, T., Miyake, K., Nakajima, K., Watanabe, T., Sakakibara, S. and Fujiwara, H. (1992) Maturation stage-associated transition and its correlation with their capacity to adhere to thymic stromal cells. J. Immunol. 149:3517–3524.

Sebzda, E., Mariathasan, S., Ohteki, T., Jones, R., Bachmann, M.F. and Ohashi, P. S. (1999) Selection of the T cell repertoire. Annu. Rev. Immunol. 17:829–874.

Sherman, L., Sleeman, J., Herrlich, P and Ponta, H. (1994) Hyaluronate receptors: key players in growth, differentiation, migration and tumor progression. Curr. Opin. Cell. Biol. 6:726–733.

Spicer, A. P., Olson, J. S. and McDonald, J. A. (1997) Molecular cloning and characterization of a cDNA encoding the third putative mammalian hyaluronan synthase. J. Biol. Chem. 272:8957–8961.

Spicer, A. P., Augustine, M. L. and McDonald, J. A. (1996) Molecular cloning and characterization of a putative mouse hyaluronan synthase. J. Biol. Chem. 271:23400–23406.

Surh, C. D. and Sprent, J. (1994) T-cell apoptosis detected *in situ* during positive and negative selection. Nature 372:100–103.

Suzuki, G., Sawa, H., Kobayashi, Y., Nakata, Y., Nakagawa, K., Uzawa, A., Sakiyama, H., Kakinuma, S., Iwabuchi, K. and Nagashima, K. (1999) Pertussis toxin-sensitive signal controls the trafficking of thymocytes across the corticomedullary junction in the thymus. J. Immunol. 162:5981–5985.

Teder, P., Bergh, J. and Heldin, P. (1995) Functional hyaluronan receptors are expressed on a squamous cell lung carcinoma cell line but not on other lung carcinoma lines. Cancer Res. 55:3908–3914.

Toole, B. P. (1997) Hyaluronan in morphogenesis. J. Int. Med. 242:35–40.

Turley, E. A. (1992) Hyaluronan and cell locomotion. Cancer and Met. Rev. 11:21–30.

Turley, E. A., Brassel, P. and Moore, D. (1990) A hyaluronan-binding protein shows a partial and temporally regulated codistribution with actin on locomoting chick heart fibroblasts. Exp. Cell Res. 187:243–249.

Turley, E. A. (1989) The role of a cell-associated hyaluronan-binding protein in fibroblast behavior. In The Biology of Hyaluronan. Evered, D. and Whelan, J., Eds. (Chichester: John Wiley and Sons), pp 121–137.

Utsumi, K., Sawada, M., Narumiya, S., Nagamine, J., Sakata, T., Iwagami, S., Kita, Y., Teraoka, H., Hirano, H., Ogata, M., Hamaoka, T. and Fujiwara, H. (1991) Adhesion of immature thymocytes to thymic stromal cells through fibronectin molecules and its significance for the induction of thymocyte differentiation. Proc. Natl. Acad. Sci. 88:5685–5689.

von Boehmer, H and Fehling, H. J. (1997) Structure and function of the pre-T cell receptor. Ann. Rev. Immunol. 15:433–452.

van Ewijk, W., Shores, E. W. and Singer, A. (1994) Crosstalk in the mouse thymus. Immunol. Today 15:214–217.

Wang, C., Thor, A. D., Moore, D. H., Zhao, Y., Kerschmann, R., Stern, R., Watson, P. H. and Turley, E. A. (1998) The overexpression of RHAMM, a hyaluronan-binding protein that regulates ras signaling, correlates with overexpression of mitogen-activated protein kinase and is a significant parameter in breast cancer progression. Clin. Cancer Res. 4:567–576.

Wang, C., Entwistle, J., Hou, G., Li, Q. and Turley, E. A. (1996) The characterization of a human RHAMM cDNA: conservation of the hyaluronan-binding domains. Gene 174:299–306.

Watanabe, K. and Yamaguchi, Y. (1996) Molecular identification of a putative human hyaluronan synthase. J. Biol. Chem. 271:22945–22948.

Watt, S. M., Thomas, J. A., Edwards, A. J., Murdoch, S. J. and Horton, M. A. (1992) Adhesion receptors are differentially expressed on developing thymocytes and epithelium in human thymus. Exp. Hematol. 20:1101–1111.

Yang, B., Yang, B. L., Savani, R. C. and Turley, E. A. (1994) Identification of a common hyaluronan binding motif in the hyaluronan binding proteins RHAMM, CD44 and link protein. EMBO J. 13:286–296.

Yang, B., Zhang, L. and Turley, E.A. (1993) Identification of two hyaluronan-binding domains in the hyaluronan receptor RHAMM. J. Biol. Chem. 268:8617–8623.

Zaballos, Á., Gutiérrez, J., Varona, R., Ardavín, C. and Márquez, G. (1999) Cutting Edge: Identification of the orphan chemokine receptor GPR-9–6 as CCR9, the receptor for the chemokine TECK. J. Immunol. 162:5671–5675.

Zaitseva, M. B., Lee, S., Rabin, R.L., Tiffany, H. L., Farber, J. M., Peden, K. W. C., Murphy, P. M. and Golding, H. (1998) CXCR4 and CCR5 on human thymocytes: Biological function and role in HIV-1 infection. J. Immunol. 161:3103–3113.

